# Characterization of cells and mediators associated with pruritus in primary cutaneous T-cell lymphomas

**DOI:** 10.1007/s10238-024-01407-y

**Published:** 2024-07-28

**Authors:** Man Hu, Jörg Scheffel, Stefan Frischbutter, Carolin Steinert, Ulrich Reidel, Max Spindler, Katarzyna Przybyłowicz, Marlena Hawro, Marcus Maurer, Martin Metz, Tomasz Hawro

**Affiliations:** 1 Institute of Allergology, Charité – Universitätsmedizin Berlin, Corporate Member of Freie Universität Berlin and Humboldt-Universität Zu Berlin, Berlin, Germany; 2 Allergology and Immunology, Fraunhofer Institute for Translational Medicine and Pharmacology ITMP, Berlin, Germany; 3 Department of Biology, Chemistry and Pharmacy, Freie Universität Berlin, Berlin, Germany; 4 Department of Dermatology, Allergology and Venereology, Charité – Universitätsmedizin Berlin, Corporate Member of Freie Universität Berlin and Humboldt-Universität Zu Berlin, Berlin, Germany; 5 Department of Dermatology, Allergology and Venereology, University Hospital Schleswig-Holstein (UKSH), Campus Lübeck, Ratzeburger Allee 160, 23538 Lübeck, Germany; 6 Institute for Inflammation Medicine, University of Lübeck, Lübeck, Germany

**Keywords:** Pruritus, Cutaneous T-cell lymphoma, Mycosis fungoides, Mast cells, Tryptase, IL-31

## Abstract

Patients with primary cutaneous T-cell lymphoma (CTCL) often experience severe and difficult-to-treat pruritus that negatively affects their quality of life (QoL). However, the mechanisms of pruritus in CTCL, including mycosis fungoides (MF), remain largely unknown, and detailed characteristics of CTCL-associated pruritus is not fully elucidated. To characterize pruritus in CTCL, cutaneous B-cell lymphoma (CBCL), and large plaque parapsoriasis (LPP), and to identify potential itch mediators involved in the pathogenesis of pruritus in CTCL patients. Clinical data and blood samples were collected from 129 healthy subjects and 142 patients. Itch intensity, QoL impairment, psychological distress, and sleep quality were assessed using validated questionnaires and instruments. Blood levels of BDNF, CCL24, GRP, IL-31, IL-33, sST2, substance P, TSLP, tryptase and total IgE were measured using ELISA or ImmunoCAP. Pruritus was prevalent in CTCL, LPP and CBCL patients, with higher prevalence and severity observed in CTCL. In CTCL, pruritus correlated with significant impairment in QoL, sleep, psychological distress. Compared to healthy controls, elevated levels of IL-31, IL-33, substance P, total IgE, tryptase, and TSLP were found in MF patients. A comparison of MF patients with and without pruritus revealed higher levels of IL-31, substance P, GRP, and CCL24 in the former. Itch intensity positively correlated with IL-31, GRP, CCL24, and tryptase levels. Pruritus significantly burdens CTCL patients, necessitating appropriate therapeutic management. Our findings suggest that various non-histaminergic mediators such as tryptase and IL-31 could be explored as novel therapeutic targets for managing pruritus in MF patients.

**Supplementary Information:**

The online version contains supplementary material available at 10.1007/s10238-024-01407-y.

## Introduction

Cutaneous T cell lymphomas (CTCL) are a heterogeneous group of lymphoproliferative disorders characterized by initial localization of malignant T-lymphocytes to the skin [1, 2]. Mycosis fungoides (MF) and Sézary syndrome (SS) are the classic subtypes of CTCL [2]. Pruritus, one of the most severe and challenging symptoms experienced by CTCL patients [3], is often chronic, long-lasting and resistant to standard treatments such as topical steroids or oral antihistamines. This severe pruritus can reduce the quality of life (QoL) and cause sleep disturbances for those affected.

Despite the high prevalence of pruritus in CTCL, there is a lack of effective treatments, mainly due to limited understanding of its underlying mechanism. In general, numerous mediators and receptors have been linked to development and maintenance of chronic pruritus, including cytokines, neuropeptides, proteases, and their respective receptors as well as ion channels and mas-related G protein-coupled receptors (MRGPR) [4–7]. In CTCL-associated pruritus, it has been postulated that interleukin (IL)-31 and other Th2 cytokines, periostin, proteases and neuropeptides are importantly involved [8, 9]. However, the data supporting these hypotheses are still sparse.

In this study, we aimed to better characterize the pruritus experienced by patients with CTCL and to explore the underlying pathomechanisms of pruritus in these patients with the aim to identify potential treatment targets. To achieve this, we assessed the concentrations of various itch-related mediators, such as neurotrophins, neuropeptides, and chemokines in the blood of MF patients and healthy individuals. Additionally, we documented the itch intensity and characteristics, and evaluated the influence of pruritus on QoL, anxiety, depression, and sleep quality.

## Materials and methods

### Study population

Overall, 142 in- and out-patients were recruited from the Dermatology Department at Charité – Universitätsmedizin Berlin for this study, including 61 with MF, 13 with SS, 32 with cutaneous B-cell lymphoma (CBCL), and 36 large plaque parapsoriasis (LPP). Not all information was available from all patients, clinical data was obtained from 123 patients (55 MF, 13 SS, 26 CBCL, 29 LPP), and blood samples were collected in the morning from 59 patients (53 MF, 6 SS). In parallel, blood samples from 129 healthy volunteers without any history of allergies, dermatological diseases, tumors, autoimmune disorders, or thyroid diseases were randomly selected from the biobank at Charité – Universitätsmedizin Berlin to serve as healthy controls for biomarker measurements. The demographics and baseline clinical characteristics are described in detail in Table 1. The number of healthy volunteers and patients assessed for each biomarker is detailed in the Supplementary Table 1.

**Table 1:** Demographics and baseline characteristics

	MF^a^ (n = 61)	SS^a^ (n = 13)	CBCL^a^ (n = 32)	LPP^a^ (n = 36)	HC^a^ (n = 129)
Gender, n (%)					
Male	36 (65.5%)	5 (38.5%)	15 (57.7%)	20 (69.0%)	48 (37.2%)
Female	19 (34.5%)	8 (61.5%)	11 (42.3%)	9 (31.0%)	81 (62.8%)
BMI (kg/m^2^)					
Median (IQR)	25.7 (23.4–28.3)	22.5 (21.6–27.3)	25 (24.1–27.1)	27 (24.2–29.6)	–
Age (years)					
Median (IQR)	68 (60–75)	70 (55–75)	55.5 (45.5–68)	70 (61–77)	41 (30–50)
Age at onset of disease (years)					
Median (IQR)	55 (45.5–62)	54.5 (52–57.8)	53 (48.3–65)	55.5 (49–59.3)	–
Duration of disease (years)					
Median (IQR)	9 (4.5–15.5)	5 (4–9)	4 (1.8–6.8)	18 (11–23.5)	–
Tumor infiltration, n (%)					
mild	31 (73.8)	2 (40)	1 (6.7)	27(100)	–
moderate	10 (23.8)	2 (40)	6 (40)	0(0)	–
severe	1 (2.4)	1 (20)	8 (53.3)	0(0)	–
BSA, n (%)					
1–20%	34 (70.8)	1 (16.7)	26 (100)	27 (96.4)	–
21–60%	9 (18.8)	0 (0)	0 (0)	1 (3.6)	–
61–100%	5 (10.4)	5 (83.3)	0 (0)	0 (0)	–
Clinical presentation of MF, n (%)					
patch	7 (13.2)	–	–	–	–
plaque	35 (66)	–	–	–	–
erythrodermic	4 (7.5)	–	–	–	–
tumor	1 (1.9)	–	–	–	–
atypical	6 (11.3)	–	–	–	–
Pruritus anytime during the course of the disease, n (%)					
yes	32 (58.2%)	12 (92.3%)	10 (38.5%)	13 (44.8%)	–^b^
no	23 (41.8%)	1 (7.7%)	16 (61.5%)	16 (55.2%)	–^b^
Current pruritus, n (%)					
yes	25 (52.1%)	3 (60.0%)	9 (34.6%)	11 (39.3%)	–^b^
no	23 (47.9%)	2 (40.0%)	17 (65.4%)	17 (60.7%)	–^b^

Pruritus anytime during the course of the disease: we asked all patients if they ever experienced pruritus during the course of their disease (yes or no). Current pruritus: It was assessed in the morning at the time of blood samples were collected by using a visual analogue scale (VAS) ranging from 0 (no itch) to 10 (worst imaginable itch)

BSA, body surface area (coefficient of the cancer proportion on skin); BMI, body mass index (calculated as weight in kilograms divided by the square of height in meters); CBCL, cutaneous B cell lymphoma; HC, healthy controls; IQR, interquartile range; LLP, large plaque parapsoriasis; MF, mycosis fungoides; SS, Sézary syndrome

^a^The reported values in the table refer to valid data only (excluding missing data)

^b^Pruritus anytime during the course of the disease and current pruritus was not assessed in healthy controls

The study was approved by the local ethics committee of the Charité – Universitätsmedizin Berlin (EA1/007/14, EA1/235/13) and adhered to the Declaration of Helsinki. Written informed consent was obtained from all participants. The diagnosis of primary cutaneous lymphoma was according to World Health Organization classification [10].

### Clinical assessments and questionnaires

The clinical characteristics of patients were recorded through a clinical interview and examination. Functional impairment of patients was assessed by the Eastern Cooperative of Oncology Group (ECOG) Performance Status Scale [11]. The severity of disease was assessed using the cancer infiltration (0–3) and body surface area (BSA) scale. The worst itch intensity during the last week and the current itch intensity in the morning at the time of blood collection, were assessed using a visual analogue scale (VAS) ranging from 0 (no itch) to 10 (worst imaginable itch). The staging of CTCL patients is classified into stages IA through IV using the TNMB system [12]. In the case of MF, stages IA, IB, and IIA are considered early-stage disease, while stages IIB through IV are categorized as late-stage disease.

The Itchy‐QoL was used as a pruritus‐specific QoL instrument [13, 14]. The Hospital Anxiety and Depression Scale (HADS) was used to assess anxiety and depression [15]. The 12-item Short-Form Health Survey (SF-12) [16], was used to assess patients’ generic Health-related QoL (HRQoL), and the Pittsburgh Sleep Quality Index (PSQI) was used to assess sleep quality and disturbances [17]. Additionally, the European Organization for Research and Treatment of Cancer (EORTC) Core Quality of Life questionnaire (QLQ-C30) version 2.0 was used to assess cancer‐specific QoL [18]. Higher scores indicate worse outcomes for the ItchyQoL, PSQI, and HADS, whereas lower scores indicate worse outcomes for the SF-12 and EORTC QOL-C30. Borderline and abnormal anxiety/depression scores are defined as HADS > 7. Poor sleep is defined as a PSQI score > 5.

In addition to the validated questionnaires, further general questions were asked. These included whether pruritus was ever experienced during the course of the disease (yes or no) and how the disease generally impairs QoL, with responses ranging from ‘not at all’, ‘a little’, ‘moderate’, ‘strong’, to ‘very strong’, whether their itch occurred at least once a day or not, whether the itch was constant or in sudden attacks, whether they have received antipruritic treatment during the course of the disease, and if so, how effective this treatment was, ranging from ‘not effective’ to ‘completely effective’. The questionnaires used and patients included in this study were part of a bigger survey on various pruritic dermatoses [19, 20].

### Blood collection and assessment of mediators

Blood was obtained by venipuncture in the morning between 8:15 a.m. and 10:00 a.m., and both serum and EDTA plasma were collected. Commercial ELISA kits were used to measure serum levels of IL-31 (R&D Systems DY2824), brain-derived neurotrophic factor (BDNF, R&D Systems DY248), substance P (R&D Systems, KGE007) and chemokine (C–C motif) ligand 24 (CCL24, R&D Systems DY343), and plasma levels of IL-33 (R&D Systems DY3625B-05), its soluble receptor sST2 (soluble suppression of tumorigenicity-2, R&D Systems DY523B05), thymic stromal lymphopoietin (TSLP, Thermo Scientific 88749788), and gastrin-releasing peptide (GRP, R&D Systems DY7847-05), all according to the manufacturer’s instructions. Serum tryptase and total IgE were measured using the ImmunoCAP System® (Phadia Laboratory Systems, Thermo Fisher Scientific Inc, Uppsala, Sweden). The decision to use either serum or plasma was based on previous investigations and experiments aimed at establishing a robust protocol.

### Statistical analysis

The statistical analysis was performed using the SciPy (version 1.8.0) in Python 3.9.12. Differences in distribution of categorical variables were tested using chi-squared test. Differences between two independent categories of parametric and non-parametric variables were tested using the two-sample t test or Mann–Whitney U test, respectively. Differences between three or more independent categories of parametric and non-parametric variables were tested using one-way analysis of variance (ANOVA, Tukey test used as a post-test) or Kruskal Wallis test (Dunn test used as a post-test), respectively. Two-way ANOVA was used to test if there is any interaction between potential mediators and gender or age. Correlations were assessed using Spearman’s rank correlation. Statistical significance was set at P < 0.05 for all tests.

## Results

### Pruritus in cutaneous lymphoma is highly prevalent and difficult to treat

**Fig. 1:**
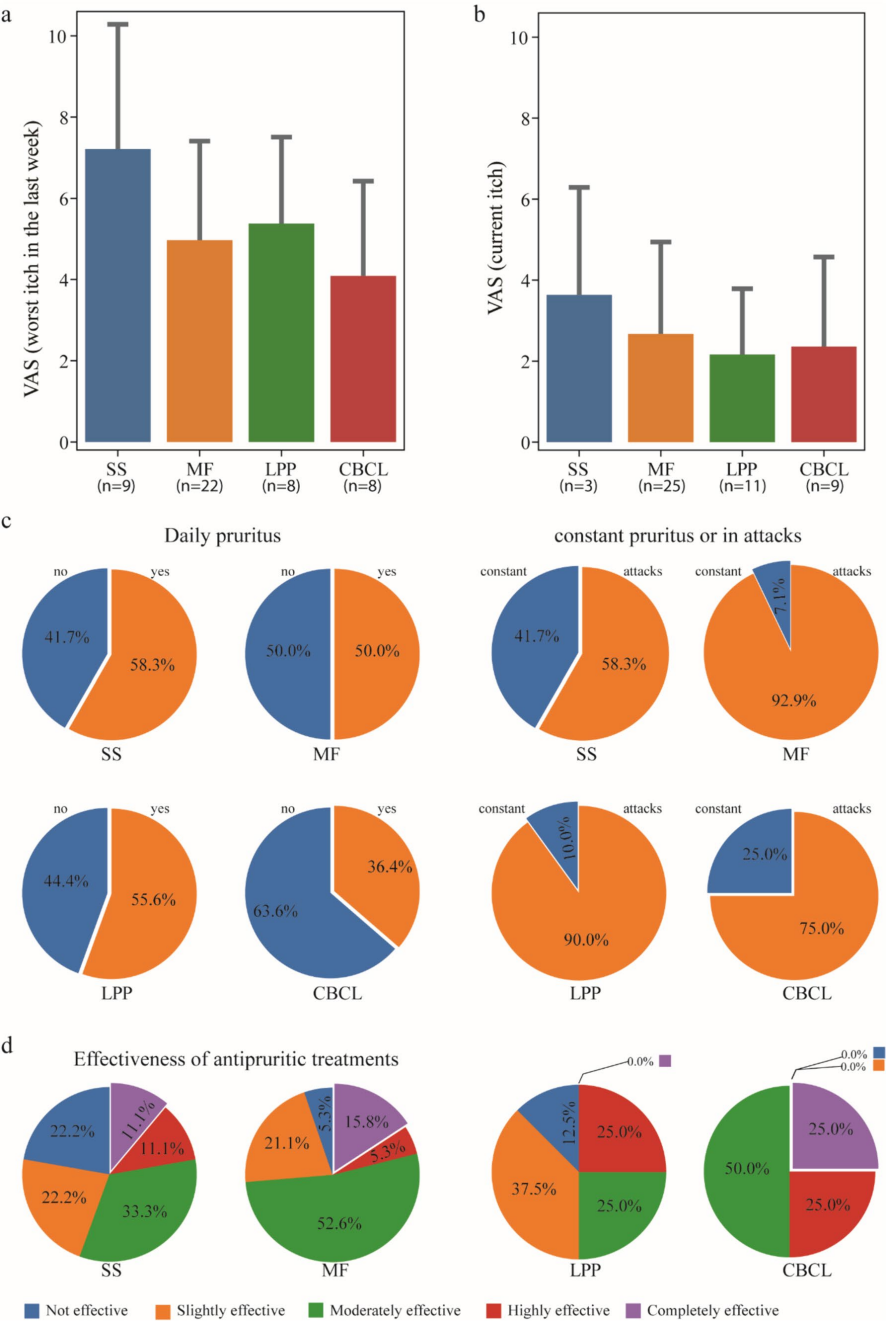
Characteristics and intensity of pruritus. **a** The intensity of worst itch in the last week in patients who reported pruritus in the last week of their respective disease. Data is presented as mean with error bars indicating standard deviation. The number below each group refers to the number of participants involved. **b** The intensity of current itch in patients who reported current pruritus in the morning during blood collection of their respective disease. Data is presented as mean with error bars indicating standard deviation. The number below each group refers to the number of participants involved. **c** The frequency (daily or not daily) and type (constant or in attacks) of itch in each group. **d** The effectiveness of antipruritic treatments in each group, the treatments mainly included antihistamines, topical steroids, and keratolytic agents. The values refer to valid data only (excluding missing data). CBCL, cutaneous B cell lymphoma; LPP, large plaque parapsoriasis; MF, mycosis fungoides; n, number; SS, Sézary syndrome; VAS, visual analogue scale.

The majority of patients with CTCL experienced pruritus. In MF, 58.2% reported pruritus during the course of their disease and 52.1% at the time of blood withdrawal, in SS patients this rate was even higher with 92.3% and 60%, respectively (Table 1). Unexpectedly, pruritus was also present, albeit to a lesser extent, in a substantial number of patients with LPP (44.8 and 39.3%) and CBCL (38.5 and 34.6%). Among those patients who reported having pruritus in the last week, the VAS intensity of worst itch within the last week was highest in SS (mean ± SD: 7.2 ± 3.3), followed by LPP (5.4 ± 2.3), MF (5.0 ± 2.5), and CBCL (4.1 ± 2.5; Fig. 1a). Current itch in patients who reported having current itch in the morning during blood collection was also most intense in SS (mean VAS: 3.6), followed by MF (2.7), CBCL (2.4), and LPP (2.2, Fig. 1b).

Pruritus occurred at least once daily in 58.3% of SS patients, 50% of MF patients, 55.6% of LPP patients, and 36.4% of CBCL patients (Fig. 1c). Patients were also asked whether their itch was constantly present or rather occurred as sudden attacks. While a large number of SS patients reported a constant itch (41.7%), sudden itch attacks were more common in MF, LPP, and CBCL (92.9%, 90%, and 75%, respectively; Fig. 1c).

Interestingly, pruritus was not restricted to lesional skin. Generalized pruritus was reported by 32.1% of MF, 22.2% of LPP, and 9.1% of CBCL patients, whereas pruritus restricted to skin lesions was present in 57.1% of MF patients, 70% of LPP patients, and 75% of CBCL patients. Overall, the majority of pruritus was reported to be symmetrical, with 77.8% in SS, 56% in MF, 50% in CBCL, and 71.4% in LPP.

Most importantly, in patients who had received antipruritic treatments, primarily including antihistamines, topical steroids, and keratolytic agents, effective treatment of pruritus, i.e. complete control of pruritus, had only been achieved in the past in 11% of SS patients, 15.8% of MF patients, 0% of LPP patients, and 25% of CBCL patients (Fig. 1d).

### Greater impairment of quality of life in MF patients with pruritus compared to those without pruritus

**Fig. 2:**
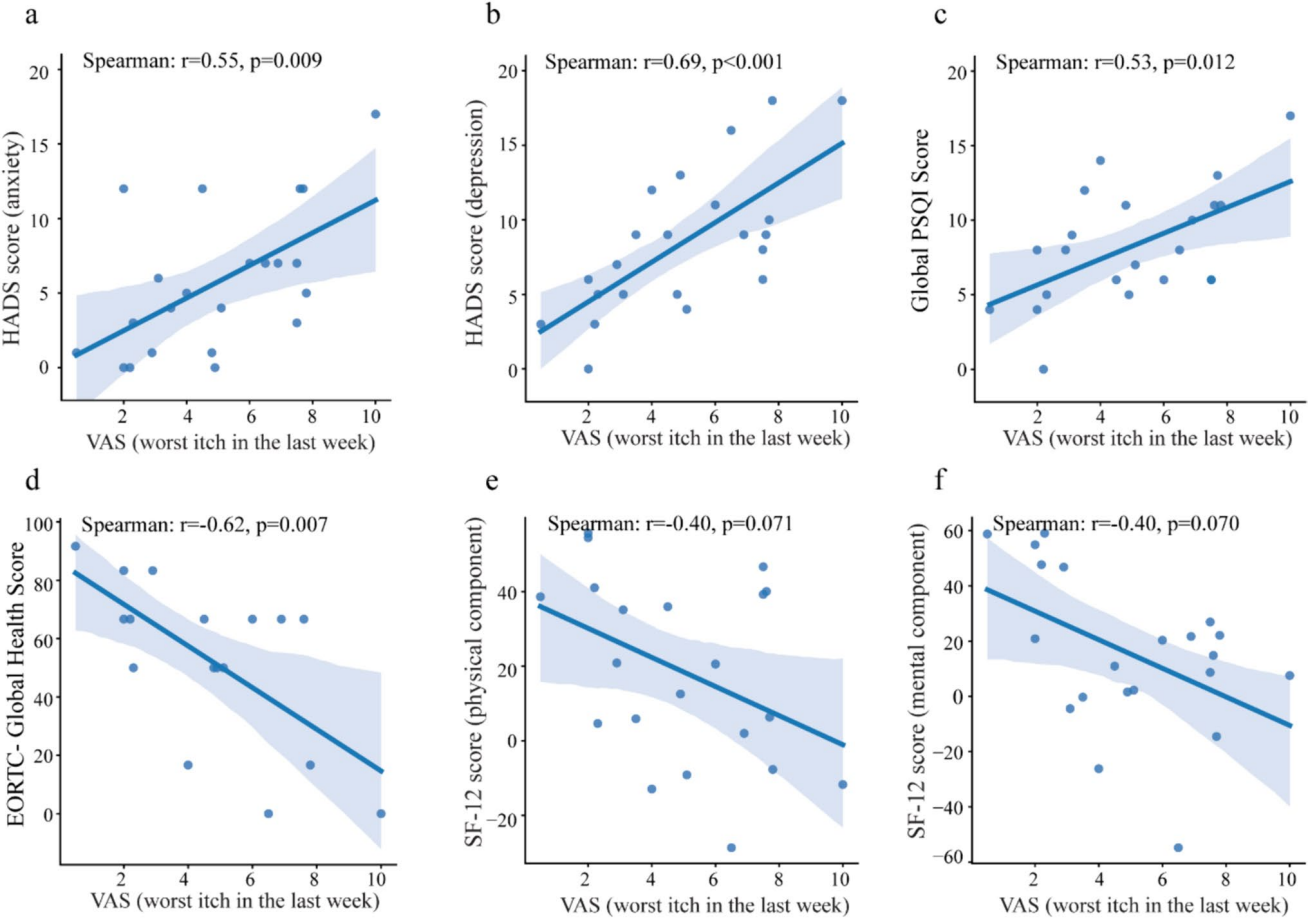
In MF patients who reported having pruritus in the last week, worst itch intensity in the last week correlates with higher levels of anxiety and depression, impairment of sleep quality and overall quality of life. The correlation of HADS-anxiety **a**, HADS-depression **b**, global sleep quality **c**, EORTC- global health score **d**, SF-12 physical component **e**, SF-12 mental component **f**, with worst itch intensity in the last week. The values refer to valid data only (excluding missing data). EORTC, European Organisation for Research and Treatment of Cancer; HADS, hospital anxiety and depression scale; MF, mycosis fungoides; PSQI, Pittsburgh Sleep Quality Index; SF-12, 12-item Short-Form Health Survey. Spearman’s rank correlation was used for analyzing the correlation between two independent variables.

Overall, the vast majority of patients with CTCL and LPP reported a negative effect of their disease on QoL, with 82.8% of MF, 83.3% of SS and 80% of LPP patients reporting at least “a little” negative effect on QoL, in CBCL patients this rate was somewhat lower, 58.3% (data not shown). To assess the impact of pruritus on QoL, we compared general and symptom-specific QoL (SF-12, EORTC, ItchyQoL), overall performance (ECOG), sleep (PSQI), and increased anxiety and depression (HADS) in patients without pruritus and those with pruritus in the last week. Despite already high rates of physical impairment, depression and limited ECOG performance, MF patients with pruritus in the last week showed significant impairment in the physical component score of the SF-12 and more depressive symptoms compared to those without pruritus (Table 2). Furthermore, the worst itch intensity within the last week in MF patients who reported having pruritus in the last week showed a strong correlation with HADS score for both anxiety and depression (r = 0.55, p = 0.009 and r = 0.69, p < 0.001, respectively, Fig. 2a, b), and poor sleep quality (PSQI score, r = 0.53, p = 0.012, Fig. 2c), especially in those with BSA > 10% and those with slight or no response to antipruritic treatment (Supplementary Table 2). Additionally, this worst itch intensity displayed a strong negative correlation with the EORTC global health score (r = −0.62, p = 0.007, Fig. 2d), and a moderate correlation with both the physical and mental component score of the SF-12 (r = −0.4, p > 0.05 for both, Fig. 2e, f). This was similar in SS patients who reported having pruritus in the last week, where worst itch intensity within the last week strongly correlated with physical and mental component score of the SF-12 (r = −0.83, p = 0.005 and r = −0.75, p = 0.020, respectively), HADS score for both anxiety and depression (r = 0.77, p = 0.014 and r = 0.69, p = 0.038, respectively), and poor sleep (PSQI score, r = 0.74, p = 0.024) (Supplementary Fig. 1).

**Table 2:** Quality of life assessments in patients with and without pruritus in the last week

	MF	SS	CBCL	LPP
ItchyQoL (total score; mean ± SD (n))				
No pruritus	–	–	–	–
Pruritus	2.4 ± 0.9(23)	3.1 ± 1.1(9)	1.8 ± 0.9(8)	2.1 ± 0.7(8)
Pittsburgh Sleep Quality Index (PSQI; global score)				
No pruritus	7.5 ± 3.9(31)	7.5 ± 3.9(4)	6.6 ± 3.9(18)	6 ± 4(21)
Pruritus	8.2 ± 3.8(23)	11.2 ± 5.9(9)	7.3 ± 5.5(8)	8.6 ± 4.4(8)
Poor sleeper (score > 5; n/total (%))				
No pruritus	19/31(61.3)	3/4(75.0)	11/18(61.1)	10/21(47.6)
Pruritus	18/23(78.3)	7/9(77.8)	4/8(50.0)	6/8(75.0)
SF-12 (physical component score, PCS; mean ± SD (n))				
No pruritus	36.7 ± 20.5(30)	23.9 ± 34.6(4)	30.7 ± 24.6(17)	25.8 ± 32.1(21)
Pruritus	18.5 ± 24.7(21) **^a^	− 4.8 ± 41.8(9)	36.9 ± 26.3(8)	17.1 ± 35.6(8)
Poor PCS (score ≤ 50; n/total (%))				
No pruritus	20/30(66.7)	3/4(75.0)	11/17(64.7)	16/21(76.2)
Pruritus	19/21(90.5)*^b^	8/9(88.9)	4/8(50.0)	7/8(87.5)
SF-12 (mental component score, MCS; mean ± SD (n))				
No pruritus	27.3 ± 30.3(30)	30.7 ± 17.5(4)	25.3 ± 31.6(17)	23.1 ± 34.4(21)
Pruritus	15.5 ± 28.6(21)	− 17 ± 46.6(9)	22.1 ± 27.9(8)	25.7 ± 27.1(8)
Poor MCS (score ≤ 42; n/total (%))				
No pruritus	16/30(53.3)	3/4(75.0)	12/17(70.6)	13/21(61.9)
Pruritus	16/21(76.2)	7/9(77.8)	6/8(75.0)	5/8(62.5)
HADS-anxiety (mean ± SD (n))				
No pruritus	5.8 ± 4(31)	3.8 ± 2.4(4)	6.3 ± 5(18)	5.7 ± 4(21)
Pruritus	5.7 ± 4.7(23)	8 ± 4.5(9)	7.4 ± 6.3(8)	7.1 ± 4.6(8)
Anxiety score > 7 (presence of at least mild anxiety; n/total (%))				
No pruritus	9/31(29.0)	0/4(0.0)	6/18(33.3)	8/21(38.1)
Pruritus	5/23(21.7)	6/9(66.7)	4/8(50.0)	4/8(50.0)
HADS-depression (mean ± SD (n))				
No pruritus	5.2 ± 4.1(31)	4.5 ± 3(4)	5.1 ± 4.1(18)	5.2 ± 3.4(21)
Pruritus	8.2 ± 4.8(23)*^a^	7.9 ± 6.1(9)	6.6 ± 6.8(8)	5.1 ± 5.1(8)
Depression score > 7 (presence of at least mild depression; n/total (%))				
No pruritus	8/31(25.8)	1/4(25.0)	5/18(27.8)	6/21(28.6)
Pruritus	12/23(52.2)*^b^	4/9(44.4)	3/8(37.5)	2/8(25.0)
EORTC-global health score (mean ± SD (n))				
No pruritus	61.8 ± 19(24)	66.7 ± 0(1)	60.9 ± 24.3(16)	65.1 ± 19.8(16)
Pruritus	52.5 ± 28.2(17)	33.3 ± 23.6(2)	66.7 ± 16.1(8)	68.1 ± 20(6)
Limited ECOG performance (n/total (%))				
No pruritus	4/28(14.3)	0/2(0.0)	2/18(11.1)	0/21(0.0)
Pruritus	8/20(40.0)*^b^	2/4(50.0)	0/8(0.0)	2/7(28.6)

Patients who declared having and no pruritus within the last week were compared. No pruritus refers to itch 0 on the VAS in the last week. Pruritus refers to itch > 0 on the VAS in the last week. Data is shown as mean ± standard deviation, unless indicated otherwise

Higher scores indicate worse outcomes for ItchyQoL, PSQI, and HADS. Lower scores indicate worse outcomes for SF-12 and EORTC QOL-C30. The reported values in the table refer to valid data only (excluding missing data)

CBCL, cutaneous B cell lymphoma; ECOG, eastern cooperative oncology group (limited ECOG performance: score > 0); EORTC QOL-C30, European organisation for research and treatment of cancer quality of life questionnaire core 30; HADS, hospital anxiety and depression scale; ItchyQol, itch-specific quality of life questionnaire; LPP, large plaque parapsoriasis; MCS, mental component score; MF, mycosis fungoides; n, number; PCS, physical component score; PSQI, Pittsburgh Sleep Quality Index; SD, standard deviation; SF-12, 12-item Short-Form Health Survey; SS, Sézary syndrome

^a^Two- sample t test or Mann–Whitney U test was used for testing the differences between two independent categories of parametric and non-parametric variables, respectively

^b^Chi-square test was used for testing whether two independent unordered binary categorical variables are related to each other

Only p values < 0.05 are shown

*p < 0.05

**p < 0.01

All four patient groups showed poor sleep quality, with 76.9% of SS, 68.5% of MF, 57.7% of CBCL and 55.2% of LPP reported a global PSQI score higher than 5, the cut-off score for “poor sleeper” [17] (data not shown). While the differences did not reach statistical significance, presence of pruritus in the last week in CTCL patients was associated with numerically worse PSQI scores (8.2 ± 3.8 vs. 7.5 ± 3.9 in MF, 11.2 ± 5.9 vs. 7.5 ± 3.9 in SS), and the rate of poor sleepers was numerically higher in MF patients with pruritus (78.3%) as compared to those without pruritus (61.3%, Table 2).

### Increased levels of pruritus- and mast cell-associated mediators in MF patients with pruritus

**Fig. 3:**
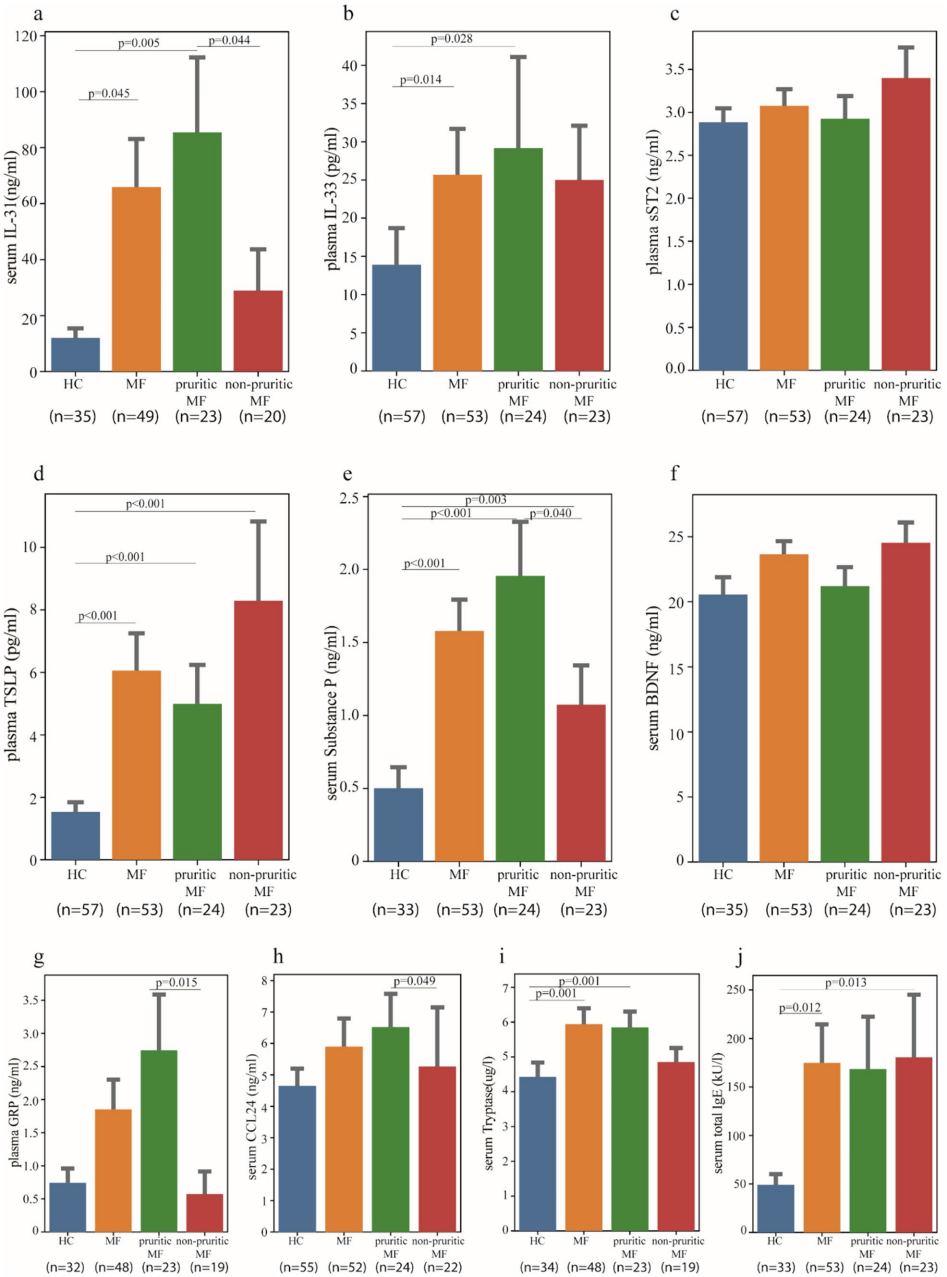
Differentially upregulated mediators in the blood of MF patients with current pruritus in the morning during blood collection compared to patients without current pruritus. The levels of serum IL-31 **a**, plasma IL-33 **b**, plasma sST2 **c**, plasma TSLP **d**, serum substance P be, serum BDNF **f**, plasma GRP **g**, serum CCL24 **h**, serum tryptase **i**, and serum total IgE **j** in healthy controls, MF patients, MF patients with and without pruritus. The values refer to valid data only (excluding missing data). The number below each group refers to the number of participants involved. Data is presented as mean with error bars indicating Standard Error of the Mean (SEM). BDNF, brain-derived neurotrophic factor; CCL24, chemokine (C–C motif) ligand 24; GRP, gastrin-releasing peptide; HC, healthy controls; IL: interleukin; MF, mycosis fungoides; n, number; sST2, soluble suppression of tumorigenicity 2; TSLP, thymic stromal lymphopoietin. Two- sample t test or Mann–Whitney U test was used for testing the differences between two independent categories of parametric and non-parametric variables, respectively.

To identify potential mediators responsible for the CTCL-associated pruritus, we assessed serum or plasma concentrations of various known itch-associated mediators and found significantly elevated levels of IL-31, IL-33, TSLP, substance P, tryptase, and total IgE in MF patients as compared to healthy controls (Fig. 3). Most strikingly, both IL-31 and substance P were not only significantly elevated in MF compared to healthy controls but also showed significantly higher levels in MF patients with current itch compared to those without, highlighting their strong association with pruritus in MF patient (Fig. 3a, e). Similarly, both GRP and, to a lesser extent, CCL24 (eotaxin-2) were found in higher concentrations in MF patients with current pruritus as compared to those without (Fig. 3g, h). Additionally, serum levels of CCL24 were significantly higher in late-stage MF patients compared to those in early-stages (p = 0.010). Compared to healthy controls, patients with pruritic MF, but not MF patients without current pruritus exhibited elevated levels of plasma IL-33 and serum tryptase (Fig. 3b, i). The levels of total IgE and TSLP were considerably increased in patients with MF, regardless of whether these patients experienced current pruritus or not (Fig. 3j, d). Plasma levels of sST2 and serum BDNF did not show any differences between the respective groups (Fig. 3c, f).

**Fig. 4:**
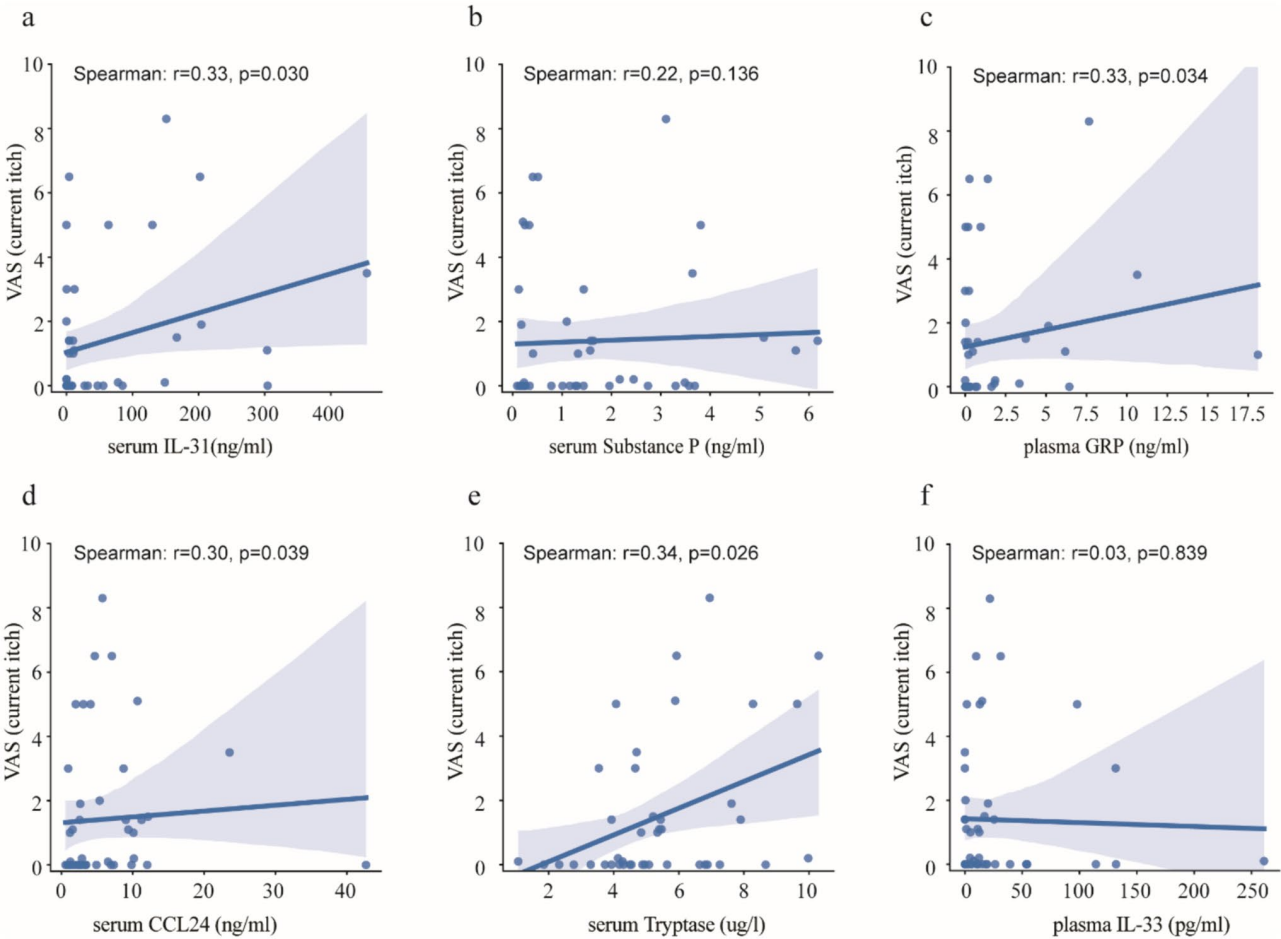
Correlation of potential itch mediators with intensity of current itch in the morning during blood collection in all MF patients. The correlation of current itch intensity with IL-31**a**, substance P **b**, GRP **c**, CCL24 **d**, tryptase **e**, IL-33 **f**. The values refer to valid data only (excluding missing data). CCL24, chemokine (C–C motif) ligand 24; GRP, gastrin-releasing peptide; IL: interleukin; MF, mycosis fungoides; VAS, visual analogue scale. Spearman’s rank correlation was used for analyzing the correlation between two independent variables.

Next, we assessed whether the levels of those mediators that were upregulated in pruritic MF patients correlate with the intensity of current pruritus. Out of these six mediators, IL-31, GRP, CCL24, and tryptase levels showed a weak, but positive correlation with the current itch intensity in MF patients (IL-31, r = 0.33, p = 0.030; GRP, r = 0.33, p = 0.034; CCL24, r = 0.30, p = 0.039; tryptase, r = 0.34, p = 0.026, respectively, significant correlations only) (Fig. 4a, c–e).

Due to the low number of blood samples from SS patients (3 SS with and 2 SS without pruritus in the morning during blood collection), we did not conduct statistical analysis. However, we observed a similar trend in SS, with higher levels of IL-31, GRP, and CCL24 in those with current pruritus compared to those without (Supplementary Fig. 2).

There was no significant interaction found between any of the measured mediators and gender or age, although MF patients and healthy controls significantly differ in terms of gender and healthy controls were significantly younger than MF patients.

## Discussion

In our study, we identified a high prevalence of pruritus and an associated considerable impact on QoL, sleep quality, physical and mental health, and psychological distress in patients with CTCL. Our results also provide evidence for a potential role of MC-associated mediators, but also neuropeptides and chemokines, in the occurrence and severity of pruritus in MF, a subtype of CTCL.

Pruritus is one of the most severe and challenging symptoms in CTCL patients [21], affecting up to 88% of all CTCL patients [22], 61% of MF patients and 94% of SS patients [23]. Our results are in line with these numbers, with a presence of pruritus in 58.2% of MF and in 92.3% of SS patients. To better characterize the impact of pruritus in CTCL on QoL, we compared itch characteristics and burden of disease in CTCL patients with or without itch to CBCL patients and patients with LPP. In CBCL, one study reported that 40% of primary CBCL patients experienced pruritus at the time of diagnosis and the pruritus was largely localized on the lesional skin [24]. In our cohort of CBCL patients, itch was present in 38.5% of patients and, indeed, only 9.1% of the patients reported generalized pruritus. Most importantly, although the worst itch intensities in CBCL were comparable to those found in MF and LPP patients, pruritus in CBCL patients had no impact on QoL, sleep or rates of anxiety and depression. This may be attributed to the majority of patients having only localized itch. Other possible explanations are that more than 60% of CBCL patients did not experience pruritus daily and that half of the patients considered the efficacy of antipruritic treatments as highly or completely effective. LPP is a non-malignant disease that shares many features with CTCL, and it is reported that about 10–35% of LPP patients progress to MF [25]. Currently, no published information is available on itch intensity and characteristics in LPP patients. In our study, 44.8% of LPP patients had pruritus, with worst itch intensities comparable to MF. However, antipruritic treatment was more often considered to be “highly effective” as compared to MF patients (25.0% in LPP vs. 5.3% in MF), and no statistically significant increase in burden of disease could be identified in LPP patients with pruritus as compared to those without.

Pruritus significantly affects CTCL patients’ QoL. Previous studies have demonstrated a strong correlation between itch and impaired QoL in CTCL patients at different disease stages [3, 26]. Our study confirms this correlation and additionally reveals that the presence of pruritus in MF also leads to higher rates of impairment in physical well-being and more depressive symptoms. Furthermore, we identified a strong correlation of worst itch intensity with signs of anxiety and depression in the HADS scoring, sleep impairment (PSQI) score, and the global health score of the EORTC in MF patients. Similar impacts of itch have been observed in other pruritus-associated conditions such as psoriasis [27], chronic pruritus of unknown origin [28], atopic dermatitis [29], and cholestatic liver diseases [30]. The impact of pruritus on QoL impairment is likely to derive directly from the bothersome symptom of pruritus itself, but also indirectly from the vicious cycle of pruritus and poor sleep or emotion [31]. Addressing QoL impairment is crucial in CTCL management, and effective itch control significantly improves treatment efficacy.

MCs are known to play a significant role in the development of various inflammatory diseases and in contributing to pruritus [32, 33]. They are also implicated in pro-tumorigenic activities, and are considered as prognostic markers and potential therapeutic targets in CTCL [34]. However, the relationship between MCs and CTCL-associated pruritus remained poorly understood. Our study identifies significant elevations in the levels of various mediators in the blood of MF patients with pruritus, suggesting a potential role of MCs in the itch associated with CTCL. These mediators, including substance P, total IgE, CCL24, tryptase, IL-31, IL-33, and GRP, although not exclusively linked to MCs, can function as either activators of MCs (substance P, IgE, IL-33, CCL24, and GRP) or as mediators released by MCs (tryptase, IL-31) [35–38], indicating a possible role of MC activation in the pathogenesis of MF-associated pruritus. It has to be noted, however, that other cell types may also be involved in CTCL-associated pruritus, either independently or in crosstalk with MCs. For example, basophils express many of the same receptors and mediators as MC and have also been implicated in chronic pruritus [39, 40], as they usually are not present in the skin of CTCL patients, a role for this cell type in CTCL-associated itch is less likely. In contrast, eosinophil infiltration is reported in CTCL and eosinophils have been associated with itch in CTCL [41] and in general to be able to contribute to chronic pruritus by interacting with MCs [42, 43]. Interestingly, many eosinophil-derived mediators, but also products from other inflammatory cells such as T cell or macrophages, can activate MC via mas-related G protein-coupled receptor X2 (MRGPRX2) [44].

The exact biological and clinical implications of these MC activators or mediators are yet to be fully elucidated. Despite significantly higher IgE levels were found in our MF patients compared to healthy controls, consistent with previous findings [45], they did not correlate with itch intensity and were similarly elevated regardless of pruritus presence. Furthermore, there are no indicators of a relevant IgE-mediated activation of MCs, i.e. the absence of specific IgE or auto-IgE antibodies, lack of wheal and flare-type skin reactions in MF patients, and no relevant response to antihistamines. Therefore, a non-IgE and non-histaminergic mechanism underlying pruritus in MF is potentially more important, and our data indicate that MRGPRX2-mediated MC activation is a likely candidate.

MRGPRX2 is a multiligand receptor that responds to various exogenous and endogenous stimuli and is almost exclusively expressed by skin MCs [44, 46]. We have recently identified an increase in MRGPRX2-positive cells in the skin of MF patients, with the majority of these cells being identified as MCs [47]. Activation of MCs through MRGPRX2 and the subsequent release of tryptase has been shown to be linked to non-histaminergic pruritus, most likely via activation of protease activated receptor 2 expressed by sensory nerves [48, 49], as observed in atopic dermatitis-related pruritus [49]. Our study demonstrates, for the first time, upregulated tryptase levels in MF patients with pruritus, but not in those without, and that tryptase levels correlated with current itch intensity. Furthermore, levels of the MRGPRX2 agonist substance P were significantly higher in pruritic MF patients compared to those without pruritus. These findings highlight the involvement of MRGPRX2-mediated MC activation in MF-related pruritus and identify potential therapeutic targets.

Furthermore, we observed elevated levels of CCL24 (eotaxin-2) in pruritic MF patients as compared to those without pruritus, with a weak but significant correlation with itch intensity. This finding is in line with a previous study that noted increased expression of CCL24 in CTCL skin [50]. CCL24 is a potent chemoattractant for eosinophils [51]. A previous study has reported increased eosinophil numbers in the skin of MF patients with severe pruritus [41], and associated blood eosinophilia with disease progression and disease-specific mortality in CTCL [52]. These observations are consistent with our results, indicating elevated CCL24 levels in late-stage MF patients. Together, these findings suggest that CCL24 and eosinophils may play a role in CTCL-associated itch, particularly among patients with poor prognosis.

IL-31, a cytokine implicated in chronic pruritus across various diseases [53], is also of interest in the context of MF-associated pruritus. While IL-31 can be produced by several cellular sources, including MCs and eosinophils [54, 55], it is thought to be primarily produced by activated Th2 cells [56]. In our study, we observed elevated serum levels of IL-31 in MF patients with pruritus and the levels correlated with itch intensity. This supports previous findings that malignant CD4 + T cells obtained from CTCL patients can produce IL-31, and its levels correlate with pruritus severity in advanced stages of the disease [57]. Moreover, increased IL-31 levels were found in the epidermis and dermal infiltrate of CTCL patients, with epidermal IL-31 levels correlating with itch severity [58], and reductions in IL-31 levels associated with improvements in pruritus [59]. These findings suggest that IL-31 could serve as a promising therapeutic target for managing CTCL-associated itch, especially in advanced cases.

Taken together, our findings underscore the importance of effectively assessing and managing pruritus in patients with CTCL. Currently, treatment options for pruritus in CTCL are very limited, highlighting the urgent need for novel therapeutic targets. Our data indicate that non-histaminergic mediators such as tryptase and IL-31 may contribute to the pathogenesis of pruritus in MF. Therapies targeting these mediators, including small molecule antagonists or monoclonal antibodies, are currently in clinical programs, and their efficacy should be tested in CTCL patients with pruritus.

## Supplementary Information

Below is the link to the electronic supplementary material.

Supplementary file1 (DOCX 834 KB)

## Data Availability

The raw data supporting the conclusions of this article will be made available by the authors, without undue reservation.

## Abbreviations

ANOVA: One-way analysis of variance
BDNF: Brain-derived neurotrophic factor
BSA: Body surface area
CBCL: Cutaneous B-cell lymphoma
CCL24: Chemokine (C–C motif) ligand 24
CTCL: Cutaneous T-cell lymphoma
ECOG: Eastern Cooperative of Oncology Group
EORTC: European Organization for Research and Treatment of Cancer
GRP: Gastrin-releasing peptide
HADS: Hospital Anxiety and Depression Scale
HRQoL: Health-related QoL
IL: Interleukin
LPP: Large plaque parapsoriasis
MCs: Mast cells
MF: Mycosis fungoides
MRGPR: Mas-related G protein-coupled receptors
MRGPRX2: Mas-related G protein-coupled receptor X2
PSQI: Pittsburgh Sleep Quality Index
QoL: Quality of life
SF-12: 12-Item Short-Form Health Survey
SS: Sézary syndrome
sST2: Soluble suppression of tumorigenicity 2
TSLP: Thymic stromal lymphopoietin
VAS: Visual analogue scale
