# Supplementary material for: Characterization of cells and mediators associated with pruritus in primary cutaneous T-cell lymphomas
Source: Clin Exp Med. 2024 Jul 28;24(1):171. doi: 10.1007/s10238-024-01407-y (PMC11284195; doi:10.1007/s10238-024-01407-y)

**Characterization of cells and mediators associated with pruritus in primary cutaneous T-cell lymphomas**

Man Hu^1,2^, Jörg Scheffel, PhD^1,2^, Stefan Frischbutter, PhD^1,2^, Carolin Steinert^1,2,3^, Ulrich Reidel^4^, Max Spindler, MD^1,2^, Katarzyna Przybyłowicz, MD^1^, Marlena Hawro, PhD^5,6^, Marcus Maurer, MD^1,2^, Martin Metz, MD^1,2*^, Tomasz Hawro, MD^5,6*^

* Both authors contributed equally

^1^ Institute of Allergology, Charité – Universitätsmedizin Berlin, Corporate Member of Freie Universität Berlin and Humboldt-Universität zu Berlin, Berlin, Germany

^2^ Fraunhofer Institute for Translational Medicine and Pharmacology ITMP, Allergology and Immunology, Berlin, Germany

^3^ Freie Universität Berlin, Department of Biology, Chemistry and Pharmacy

^4^ Department of Dermatology, Allergology and Venereology, Charité – Universitätsmedizin Berlin, Corporate Member of Freie Universität Berlin and Humboldt-Universität zu Berlin, Berlin, Germany

^5^ Department of Dermatology, Allergology and Venereology, University Hospital Schleswig-Holstein (UKSH), Campus Lübeck, Lübeck, Germany

^6^ Institute for Inflammation Medicine, University of Lübeck, Lübeck, Germany

**Corresponding author:**

Tomasz Hawro, M.D.

Department of Dermatology, Allergology and Venereology

University Hospital Schleswig-Holstein (UKSH), Campus Lübeck

Lübeck, Germany

Ratzeburger Allee 160, 23538 Lübeck

T +49 451-500 41602, Fax: +49 451-500 41628

[tomasz.hawro@uksh.de](mailto:tomasz.hawro@uksh.de)

**Materials and Methods**

| **Supplementary Table 1. The number of healthy controls and patients assessed for circulating biomarkers** | | | | | | | | | | |  |
| --- | --- | --- | --- | --- | --- | --- | --- | --- | --- | --- | --- |
|  | IL-31 | SP | tIgE | Tryptase | sST2 | IL-33 | TSLP | BDNF | CCL24 | GRP | |
| HC, n | 35 | 33 | 33 | 34 | 57 | 57 | 57 | 35 | 55 | 32 | |
| MF, n | 49 | 53 | 53 | 48 | 53 | 53 | 53 | 53 | 52 | 48 | |
| SS, n | 6 | 6 | 6 | 6 | 6 | 6 | 6 | 6 | 6 | 6 | |
| Abbreviations: BDNF, Brain-derived neurotrophic factor; CCL24, Chemokine (C-C motif) ligand 24; GRP, Gastrin-releasing peptide; HC, Healthy controls; IL: Interleukin; MF, Mycosis fungoides; n, Number; SP, Substance P; SS, Sézary syndrome; sST2, Soluble suppression of tumorigenicity 2; tIgE, Total IgE; TSLP, Thymic stromal lymphopoietin. | | | | | | | | | | |  |

| **Supplementary Table 2. The correlation between worst itch intensity in the last week and anxiety and depression, impairment of sleep quality and overall quality of life in subgroup of MF patients** | | | | | | |
| --- | --- | --- | --- | --- | --- | --- |
|  | HADS score (anxiety) | HADS score (depression) | Global PSQI score | EORTC-Global health score | SF-12 (physical component) | SF-12 (mental component) |
| **MF patietns with late-stage (n=5)** | | | | | | |
| Correlation p value | 0.262 | 0.8 | <0.001 | 0.8 | 0.2 | 0.8 |
| Correlation r | 0.74 | 0.20 | 1 | -0.2 | -0.8 | -0.2 |
| **MF patietns with BSA>10% (n=22)** | | | | | | |
| Correlation p value | 0.014 | 0.009 | 0.016 | 0.023 | 0.061 | 0.041 |
| Correlation r | 0.64 | 0.67 | 0.63 | -0.65 | -0.53 | -0.57 |
| **MF patietns with no or a little response to antipruritic treatment (n=5)** | | | | | | |
| Correlation p value | 0.188 | 0.054 | 0.037 | <0.001 | 0.037 | 0.873 |
| Correlation r | 0.7 | 0.87 | 0.9 | -1 | -0.9 | -0.1 |
| Abbreviations: EORTC, European Organisation for Research and Treatment of Cancer; HADS, Hospital anxiety and depression scale; MF, Mycosis fungoides; n, Number; PSQI, Pittsburgh Sleep Quality Index; SF-12, 12-item Short-Form Health Survey. Higher scores indicate worse outcomes for ItchyQoL, PSQI, and HADS. Lower scores indicate worse outcomes for SF-12 and EORTC QOL-C30. The reported values in the table refer to valid data only (excluding missing data). Spearman's rank correlation was used for analyzing the correlation between two independent variables. Statistical significance was set at P < 0.05. | | | | | | |

**FIGURE LEGENDS**

**Supplementary Fig.1 In SS patients who reported having pruritus in the last week, worst itch intensity in the last week correlates with worse generic Health-related QoL, higher levels of anxiety and depression, and impairment of sleep quality**

The correlation of HADS-anxiety (a), HADS-depression (b), global sleep quality (c), SF-12 physical component (d), SF-12 mental component (e), with worst itch intensity in the last week. The values refer to valid data only (excluding missing data). Excluded EORTC- global health score as not enough valid data to perform statistical analyses. Abbreviations: EORTC, European Organisation for Research and Treatment of Cancer; HADS, Hospital anxiety and depression scale; PSQI, Pittsburgh Sleep Quality Index; SF-12, 12-item Short-Form Health Survey; SS, Sézary syndrome. Spearman rank correlation test was used for analyzing the correlation between two independent variables.

**Supplementary Fig. 2 Differentially upregulated mediators in the blood of SS patients with current pruritus in the morning during blood collection compared to patients without current pruritus**

The levels of serum IL-31 (a), plasma IL-33 (b), plasma sST2 (c), plasma TSLP (d), serum Substance P (e), serum BDNF (f), plasma GRP (g), serum CCL24 (h), serum tryptase (i), and serum total IgE (j) in healthy controls, SS patients, SS patients with and without pruritus. The values refer to valid data only (excluding missing data). The number below each group refers to the number of participants involved. Data is presented as mean with error bars indicating Standard Error of the Mean (SEM). Abbreviations: BDNF, Brain-derived neurotrophic factor; CCL24, Chemokine (C-C motif) ligand 24; GRP, Gastrin-releasing peptide; HC, Healthy controls; IL: Interleukin; n, Number; SS, Sézary syndrome; sST2, Soluble suppression of tumorigenicity 2; TSLP, Thymic stromal lymphopoietin.

**Supplementary Fig. 1**
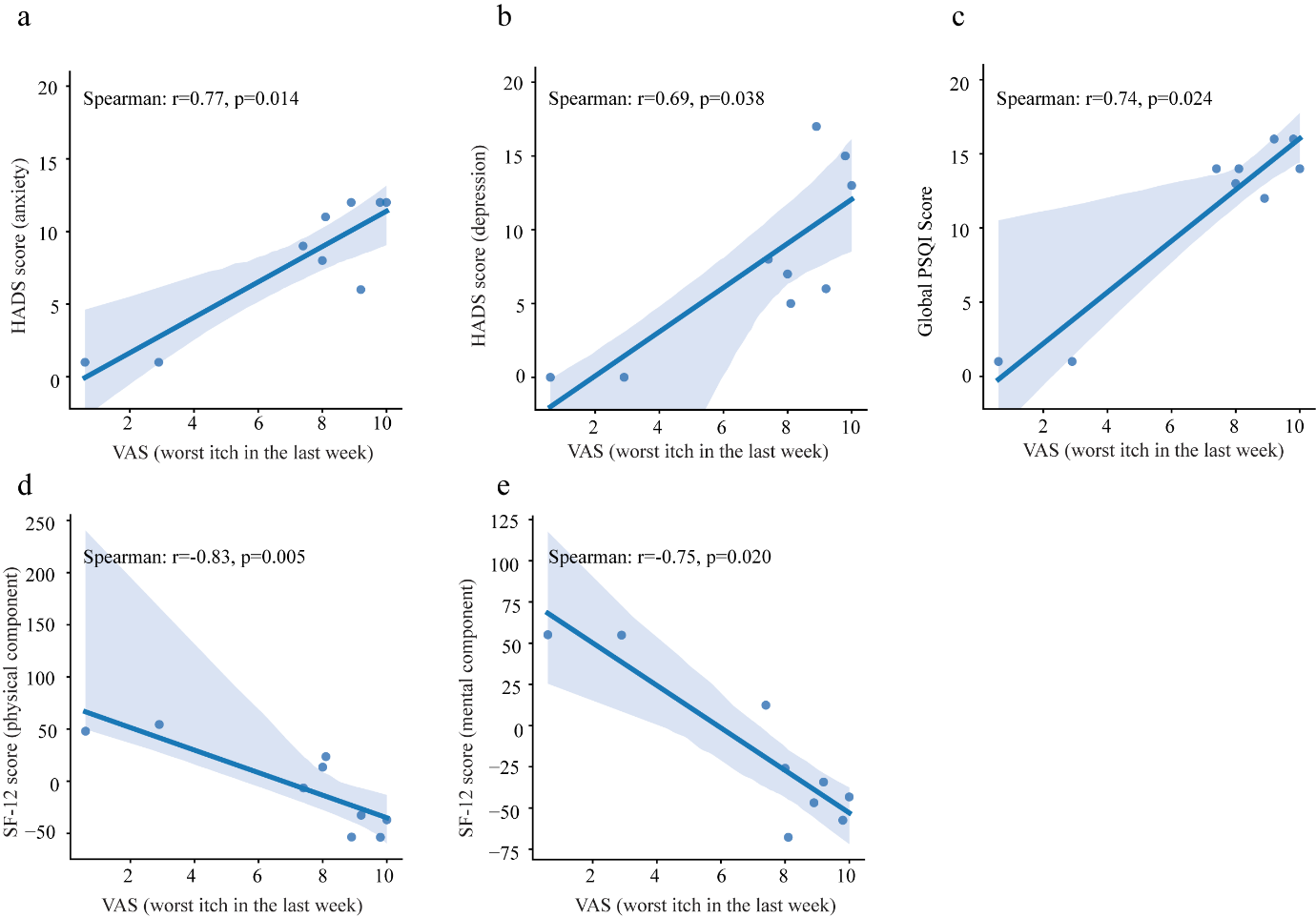


**Supplementary Fig. 2**
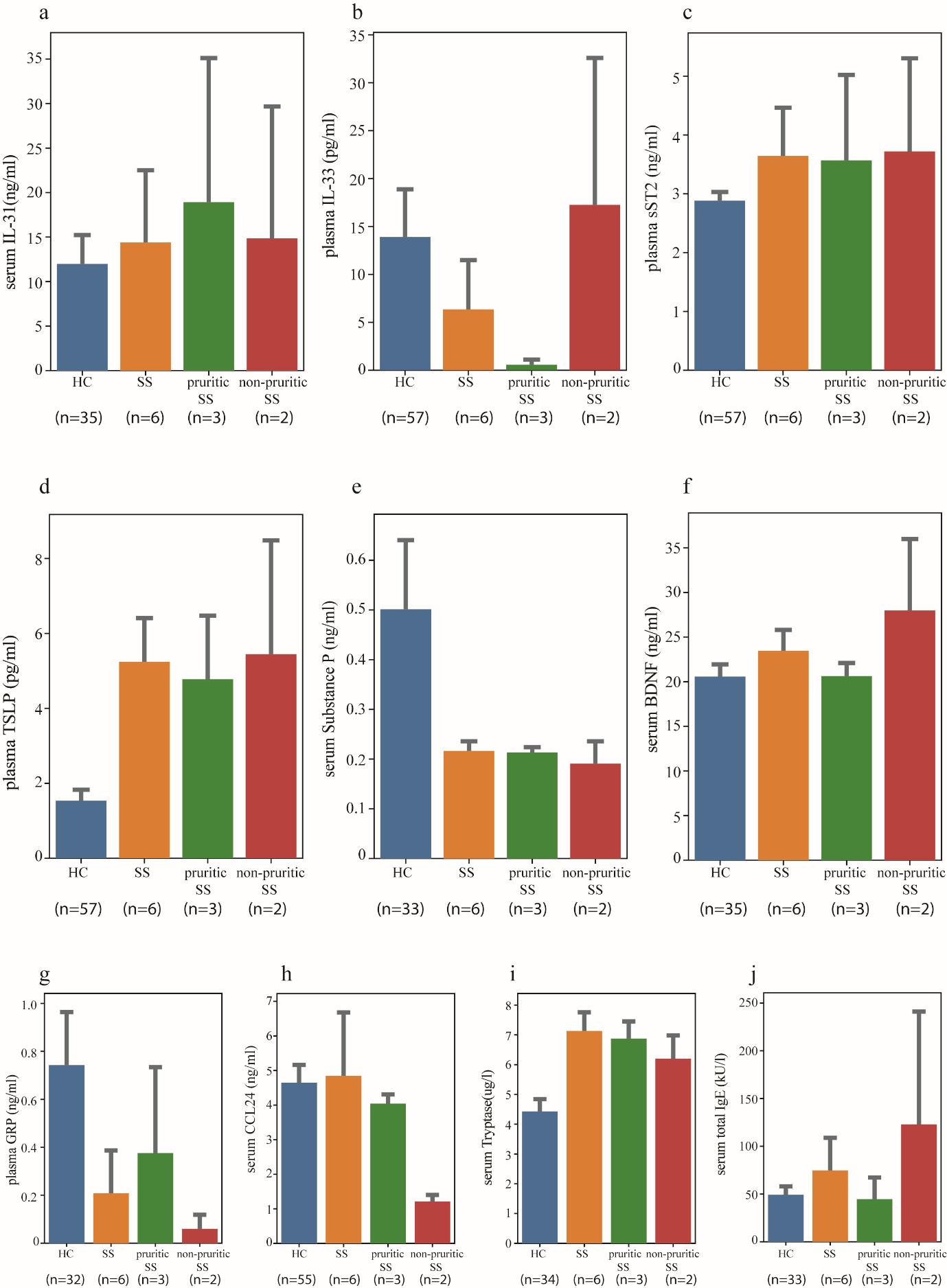

Supplement: Supplementary file 1 — Supplementary file1 (DOCX 834 KB) [file 10238_2024_1407_MOESM1_ESM.docx]
